# Structure-based computational screening of 470 natural quercetin derivatives for identification of SARS-CoV-2 M^pro^ inhibitor

**DOI:** 10.7717/peerj.14915

**Published:** 2023-03-14

**Authors:** Abd. Kakhar Umar, James H. Zothantluanga, Jittima Amie Luckanagul, Patanachai Limpikirati, Sriwidodo Sriwidodo

**Affiliations:** 1 Department of Pharmaceutics and Pharmaceutical Technology, Faculty of Pharmacy, Padjadjaran University, Sumedang, Jawa barat, Indonesia; 2 Department of Pharmaceutics and Industrial Pharmacy, Faculty of Pharmacy, Chulalongkorn University, Bangkok, Thailand; 3 Department of Pharmaceutical Sciences, Faculty of Pharmacy, Dibrugarh University, Assam, India; 4 Department of Food and Pharmaceutical Chemistry, Faculty of Pharmacy, Chulalongkorn University, Bangkok, Thailand

**Keywords:** Quercetin, SARS-CoV-2, COVID-19, Main protease, *In-silico*

## Abstract

Coronavirus disease 2019 (COVID-19) is a global pandemic infecting the respiratory system through a notorious virus known as the severe acute respiratory syndrome coronavirus 2 (SARS-CoV-2). Due to viral mutations and the risk of drug resistance, it is crucial to identify new molecules having potential prophylactic or therapeutic effect against SARS-CoV-2 infection. In the present study, we aimed to identify a potential inhibitor of SARS-CoV-2 through virtual screening of a compound library of 470 quercetin derivatives by targeting the main protease—Mpro (PDB ID: 6LU7). The study was carried out with computational techniques such as molecular docking simulation studies (MDSS), molecular dynamics (MD) simulations, and molecular mechanics generalized Born surface area (MMGBSA) techniques. Among the natural derivatives, compound 382 (PubChem CID 65604) showed the best binding affinity to Mpro (−11.1 kcal/mol). Compound 382 interacted with LYS5, TYR126, GLN127, LYS137, ASP289, PHE291, ARG131, SER139, GLU288, and GLU290 of the Mpro protein. The SARS-CoV-2 Mpro-382 complex showed acceptable stability during the 100 ns MD simulations. The SARS-CoV-2 Mpro-382 complex also showed an MM-GBSA binding free energy value of -54.0 kcal/mol. The binding affinity, stability, and free energy results for 382 and Mpro were better than those of the native ligand and the standard inhibitors ledipasvir and cobicistat. The conclusion of our study was that compound 382 has the potential to inhibit SARS-Cov-2 Mpro. However, further investigations such as *in-vitro* assays are recommended to confirm its *in-silico* potency.

## Introduction

Coronavirus disease 2019 (COVID-19) is a global pandemic infecting the respiratory system through a notorious virus known as the severe acute respiratory syndrome coronavirus 2 (SARS-CoV-2) (Nadjib, 2020). COVID-19 initially emerged from Wuhan, China, in December 2019 (Zothantluanga et al., 2021b). As of 14th June 2022, COVID-19 has infected 532,887,531 million people and has killed 6,307,201 million people (https://covid19.who.int/). Many vaccines have been developed for COVID-19 (Polack et al., 2020; Sadoff et al., 2021; Knoll & Wonodi, 2021), and as many as 11,854,673,610 vaccine doses have been administered across the globe (https://covid19.who.int/). However, viral mutations have resulted in the emergence of multiple variants of SARS-CoV-2 with increased transmission rates and that are more infectious, and more resistant to the available therapeutic and prophylactic treatments (Mistry et al., 2022). Moreover, COVID-19 affects not only the respiratory system but also adversely affects other physiological functions (Elfiky, 2020). Therefore, research is ongoing in all possible areas to discover new therapies for the early diagnosis and treatment of COVID-19 (Tsang et al., 2021). Among the different approaches available, natural screening products for the potential identification of inhibitors of SARS-CoV-2 is a popular approach  (Nugraha et al., 2020; Adeleye et al., 2021; Wang et al., 2020). This is because secondary metabolites of plants have shown promising antiviral activities (Denaro et al., 2020). Therefore, it is logical to explore natural product databases in search of potential inhibitors of SARS-CoV-2.

The main protease (Mpro) of SARS-CoV-2 is one of the most popular drug targets. SARS-CoV-2 Mpro breaks down larger viral polyproteins produced during the initial viral replication process. These polyproteins undergo proteolytic cleavage at 11 sites which the Mpro mediates. Following this, Mpro breaks down the polyproteins into different non-structural proteins essential for the multiplication of viruses  (Umar et al., 2022). Natural products have always harboured various phytochemicals with potent inhibitory activity against SARS-CoV-2. In the early days of the pandemic, *in-silico* techniques were mainly used to screen natural compounds against SARS-CoV-2 Mpro. To name a few, researchers have identified andrographolide, green tea polyphenols, toxins, and cannabinoids as inhibitors of SARS-CoV-2 by targeting the Mpro (Enmozhi et al., 2020; Ghosh et al., 2021; Raj et al., 2021; Ibrahim et al., 2022). Research trends indicate that phytochemicals have the potential as a natural reservoir for potent inhibitors of SARS-CoV-2. Phytochemicals and herbal extracts showed promising results when their inhibitory potential against SARS-CoV-2 was investigated with *in-vitro* techniques (Zannella et al., 2021; Pasquereau et al., 2021; Liu et al., 2021).

Quercetin is a dietary flavonoid abundantly found in fruits and vegetables. Quercetin is also available commercially as a dietary supplement (Andres et al., 2018). Quercetin is a well-known bioactive compound with diverse biological activities such as antioxidant, anti-inflammatory, anti-Alzheimer, anti-Parkinson, anticancer, antiulcer, antibacterial, anti-hyperlipidemia, and antiviral. Quercetin was also reported to be useful in treating cardiovascular diseases, metabolic disorders, renal diseases, allergies, asthma, hay fever, and hives (Anand David, Arulmoli & Parasuraman, 2016; Umar, Kelutur & Zothantluanga, 2021; Umar & Zothantluanga, 2021; Patala, Anggi & Butar-butar, 2022). Quercetin exhibits antiviral activities against a wide array of viruses (Di Petrillo et al., 2022; Umar, 2021). Moreover, clinical trials have already demonstrated that quercetin reduces the severity of COVID-19 (Di Pierro et al., 2021; Önal et al., 2021). As quercetin has already been proven to be a promising COVID-19 therapy, we aim to identify the most potent and safe form of natural quercetin as inhibitor of SARS-CoV-2 through virtual screening of a compound library of quercetin derivatives by targeting Mpro. To achieve this, computational techniques such as molecular docking simulation studies (MDSS), molecular dynamics (MD) simulations, and molecular mechanics generalized Born surface area (MM-GBSA) techniques were used to identify a quercetin derivative with inhibitory potential against SARS-CoV-2 Mpro.

## Methods

### Retrieval of ligands and their preparation

Ligands used in the study were obtained from the PubChem database with the keyword ‘Quercetin derivates’. The search showed results of 700 molecules of quercetin and its derivatives. All ligands were downloaded in a single file as the structure data file format. Each file was split with OpenBabel (v2.3.1). Following this, duplicates of each ligand were removed with the command “–unique” in Open Babel. After removing duplicates, the remaining 470 unique quercetin derivatives were saved in the protein data bank (PDB) file format. The energies of the ligands were minimized with the “obminimize” command with the application of the ‘MMFF94s’ force field using OpenBabel.

### Protein preparation

Mpro was obtained from the RCSB-PDB website (https://www.rcsb.org/structure/6lu7). The protein has a PDB ID of 6LU7 (Umar, 2021). The native ligand and the protein were separated with the Discovery Studio 2021 Client (DS) (BIOVIA, San Diego, CA, USA). The protein was optimized with AutoDock Tools to remove the water, regulate the charges (Kollman charges), and add polar hydrogen (Forli et al., 2016). The protein was converted into the PDBQT format and was saved for future use.

### Dockthor virtual screening

Screening of ligands was carried out with the DockThor server (https://dockthor.lncc.br/v2/index.php). The target protein (6LU7) was selected through the ‘protein’ menu tab. The virtual screening was carried out per 200 ligands (limit of DockThor) without a cofactor. The grid box was adjusted to coordinates *x* =  − 10.72, *y* = 12.41, *z* = 68.81 based on the experimental pose and position of native ligand, with a size of 40 × 40 × 40 Å, discretization of 0.42, 500,000 evalutations, population size of 750, initial seed of -1,985, 12 runs, and checked soft docking option. The screening results of the test ligands were compared with the ligand references (COVID-19 repurposing drug dataset on DockThor) to identify promising test ligands worthy of further studies. Protonation assignment and hydrogen-bond optimization were performed using ProtAssign and PROPKA (Guedes et al., 2021). The RMSD of docked native ligand was determined using PyMOL to validate the docking method used. Top compounds were ranked by the docking score.

### Molecular dynamics simulations

MD simulation studies were carried out according to the protocols described in our previous studies (Pasala et al., 2022b). The operating system of the computational setup was Linux Ubuntu20.04.1 LTS 64-bit. The graphic card, RAM, and processor were NVIDIA Quadro P2200, 16 GB, and Intel Xeon(R) W-2223 @3.60 GHz octa-core. Desmond on Maestro Schrödinger2021–2 was the software package for MD simulation studies (Schrodinger, 2020a; Schrodinger, 2020b; Bowers et al., 2006). Briefly, the protein-ligand complex obtained from the output of the molecular docking studies was used for the MD simulation studies. Protonation of the complex was assigned using PROPKA at pH 7. The protein-ligand complex was placed in a simple point charge water modeled cubic box (10 Å). Sodium ions (Na^+^) and chloride ions (Cl^−^) were added to mimic physiological conditions. To neutralize the charge, the salt ion was set at 0.15 M with the addition of counter ions. The temperature and pressure were adjusted to 300K and 1.63 bar using NPT essemble, respectively. With the OPLS_2005 force field application, the MD simulations were carried out for 100 ns. The recording intervals for energy and trajectory were set at 1.2 ps and 20 ps, respectively. For pressure control, the Martyna-Tuckerman-Klein chain coupling scheme was employed, and for temperature monitoring, the Nose Hoover chain coupling method was used (Martyna, Klein & Tuckerman, 1992).

### MM-GBSA binding free energy calculations

According to the protocols described in our previous studies (Umar et al., 2022), the protein-ligand complexes’ binding free energies (BFE) were carried out with the MM-GBSA approach. This calculation was only performed on top compound obtained from molecular docking and native ligand. Briefly, the Desmond module of the Maestro-Schrodinger 2021-3 was used to generate the trajectories of the MD simulations (*-cms.out). The Schrodinger’s thermal_mmgbsa.py script was executed to calculate the average MM-GBSA BFE in the MD trajectories (Duarte et al., 2021; Mahmoud et al., 2021). The MD trajectories were split into individual frames of 1001 snapshots. Each snapshot represents the input for computing the MM-GBSA BFE. Equation (1) was used to calculate the MM-GBSA BFE. The BFE is represented as ΔGbind, and the free energies of the ligand, receptor, and complex are represented as Gligand, Greceptor, and Gcomplex, respectively. Individual energies such as coulomb, covalent, Hbond, Lipo, Packing, SelfCont, Solv GB, Solv SA, and vdW were used to calculate the energies of each of the Gcomplex, Greceptor, and Gligand respectively. Schrödinger’s thermal MM-GBSA script (https://www.schrodinger.com/scriptcenter) was used to obtain the MM-GBSA values, which were then used to calculate the MM-GBSA BFE of the protein-ligand complexes (Li et al., 2011; De Campos, Palermo & Conda-Sheridan, 2021).

(1) \begin{eqnarray*}\Delta \text{Gbind}=\text{Gcomplex}-\text{Greceptor}-\text{Gligand}.\end{eqnarray*}

### *In-silico* ADMET screening

The physicochemical properties, pharmacodynamics, pharmacokinetic parameters, and bioavailability of the most potent compound was studied with the SwissADME web tool (Daina, Michielin & Zoete, 2017). The ProTox-II web server was used to compute the median lethal dose (LD_50_), toxicity class, and toxicity end points of the most potent compound (Banerjee et al., 2018).

## Results

### Virtual screening

**Figure 1:**
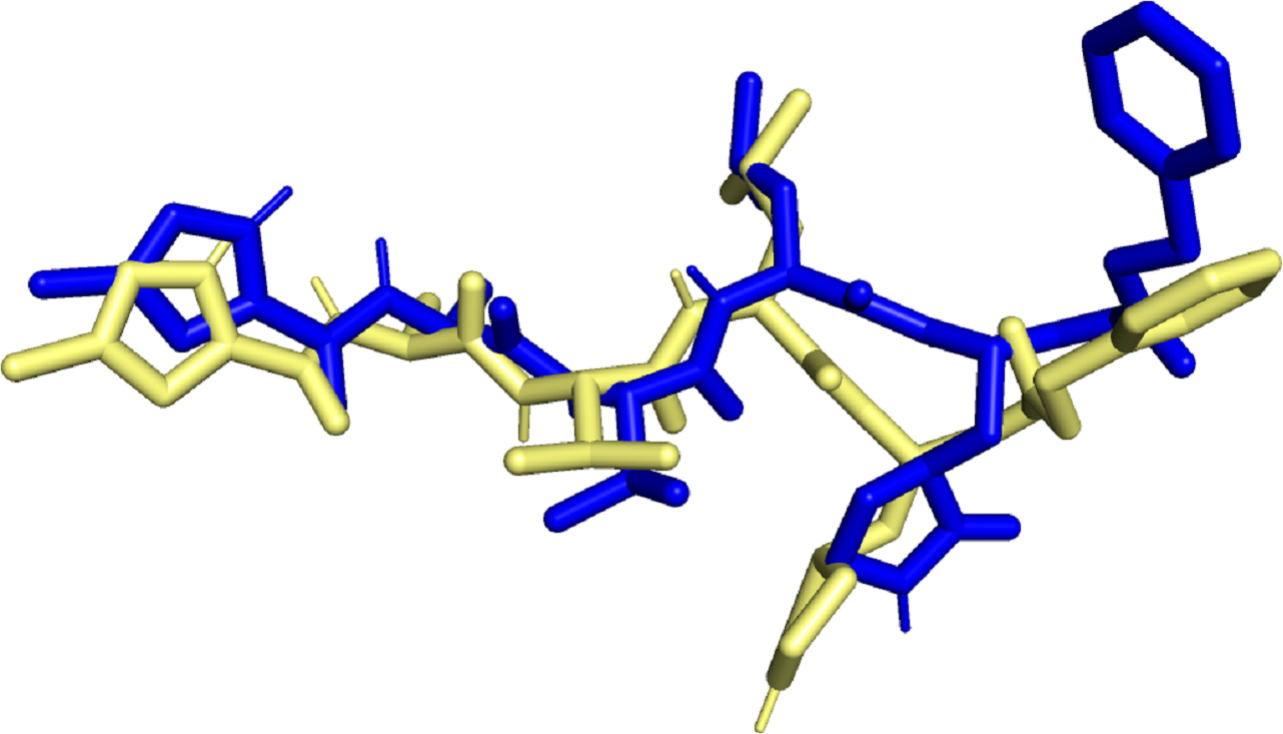
Superimposed native ligand (Blue) over its experimental pose (Yellow).

Molecular docking simulation studies were carried out for 470 quercetin derivatives. In addition to this, the native ligand of 6LU7, cobicistat (anti-HIV drug), and ledipasvir (anti-hepatitis C) were used as positive controls. These compounds were also subjected to molecular docking using the DockThor server. The docking score values of the positive control were used as the benchmark, and only the top quercetin derivative with lowest docking score values than the positive control were selected for further studies. The docked native ligand superimposed over the experimental pose can be seen in Fig. 1. The RMSD obtained was 1.467Å with docking score value of −8.7 kcal/mol, indicating that the docking method used was valid.

**Figure 2:**
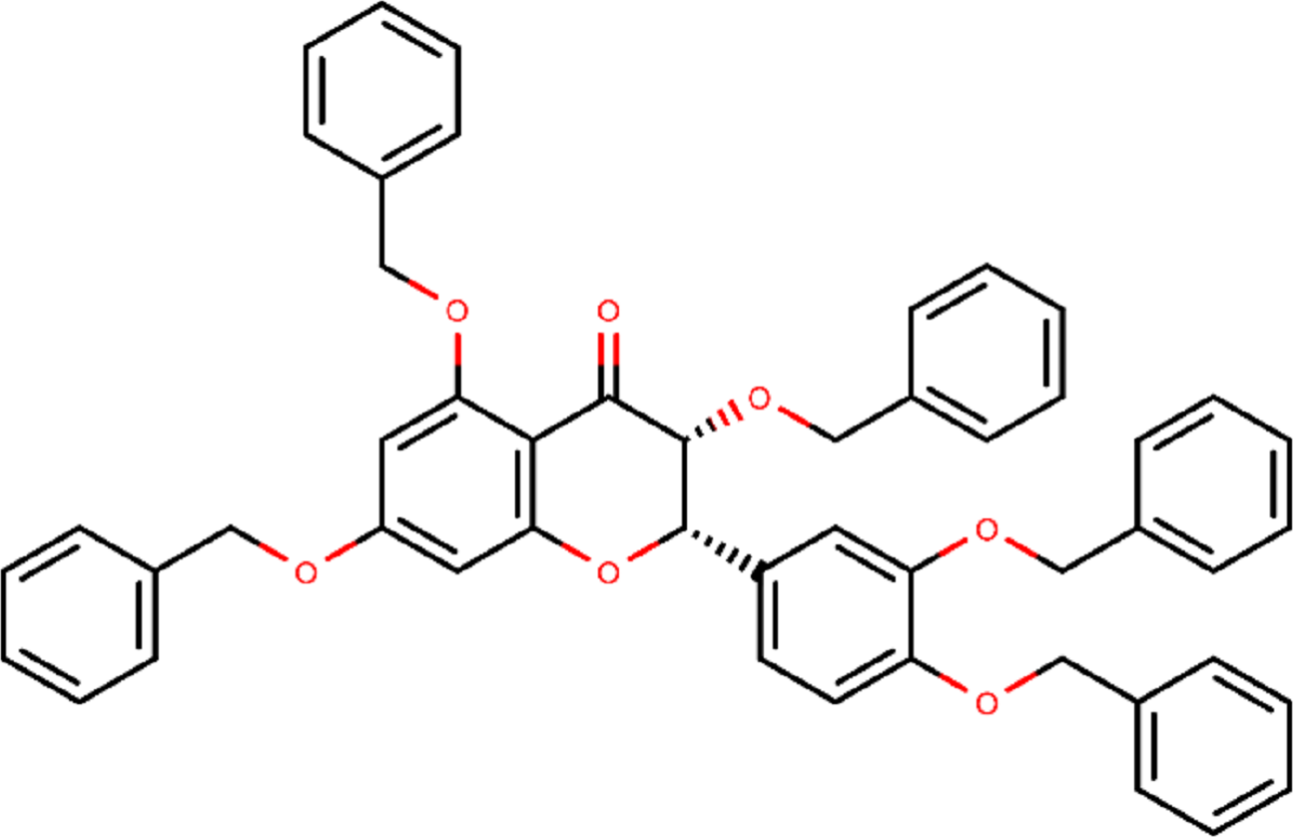
Chemical structure of compound 382 (PubChem CID 65604).

The results of the molecular docking simulation studies for the positive control and the best quercetin derivative are given in Table 1. Among the positive control, ledipasvir (−10.6 kcal/mol) showed the lowest docking score value towards the active binding site of SARS-CoV-2 Mpro, followed by cobicistat (−9.5 kcal/mol) and the native ligand (−8.7 kcal/mol). Among all the quercetin derivatives, 382 (−11.1 kcal/mol) showed the lowest docking score value (Fig. 2). The docking score value of quercetin derivative 328 is lower than the three positive controls used in the study. This indicates that 382 has a better binding affinity for SARS-CoV-2 Mpro than the native ligand, ledipasvir, and cobicistat. Compound 382 showed a promising result and was subjected to further studies.

**Figure 3:**
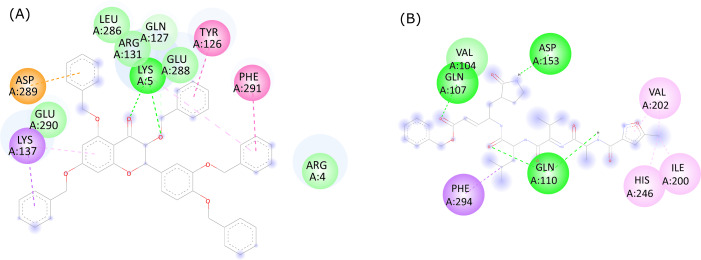
2D ligand interactions between (A) quercetin derivative 382 and (B) the native ligand with the active site residues of SARS-CoV-2 Mpro.

The molecular interactions between 382, the native ligand, and the active site residues of SARS-CoV-2 were analyzed with the Discovery Studio Visualizer v.20.1.0.19295 software. Based on the current binding pose (Fig. 3A), 382 was found to interact with LYS5 (two conventional hydrogen bonds), GLN127 (one carbon hydrogen bond), TYR126 (one hydrophobic interaction), GLN127 (one carbon-hydrogen bond), LYS137 (two hydrophobic interactions), ASP289 (one electrostatic interaction), PHE291 (one hydrophobic interaction). Van der Waals interactions were also shown by ARG131, SER139, GLU288, and GLU290. On the other hand, the native ligand (Fig. 3B) showed conventional hydrogen bonds with GLN107 (*n* = 1), GLN110 (*n* = 2), and ASP153 (*n* = 1). The native ligand also showed hydrophobic interactions with ILE200 (*n* = 1), VAL202 (*n* = 2), HIS246 (*n* = 1), PHE294 (*n* = 1). The bond type and bond length of 382 and Mpro are provided in Table 2.

### MD simulations

**Figure 4:**
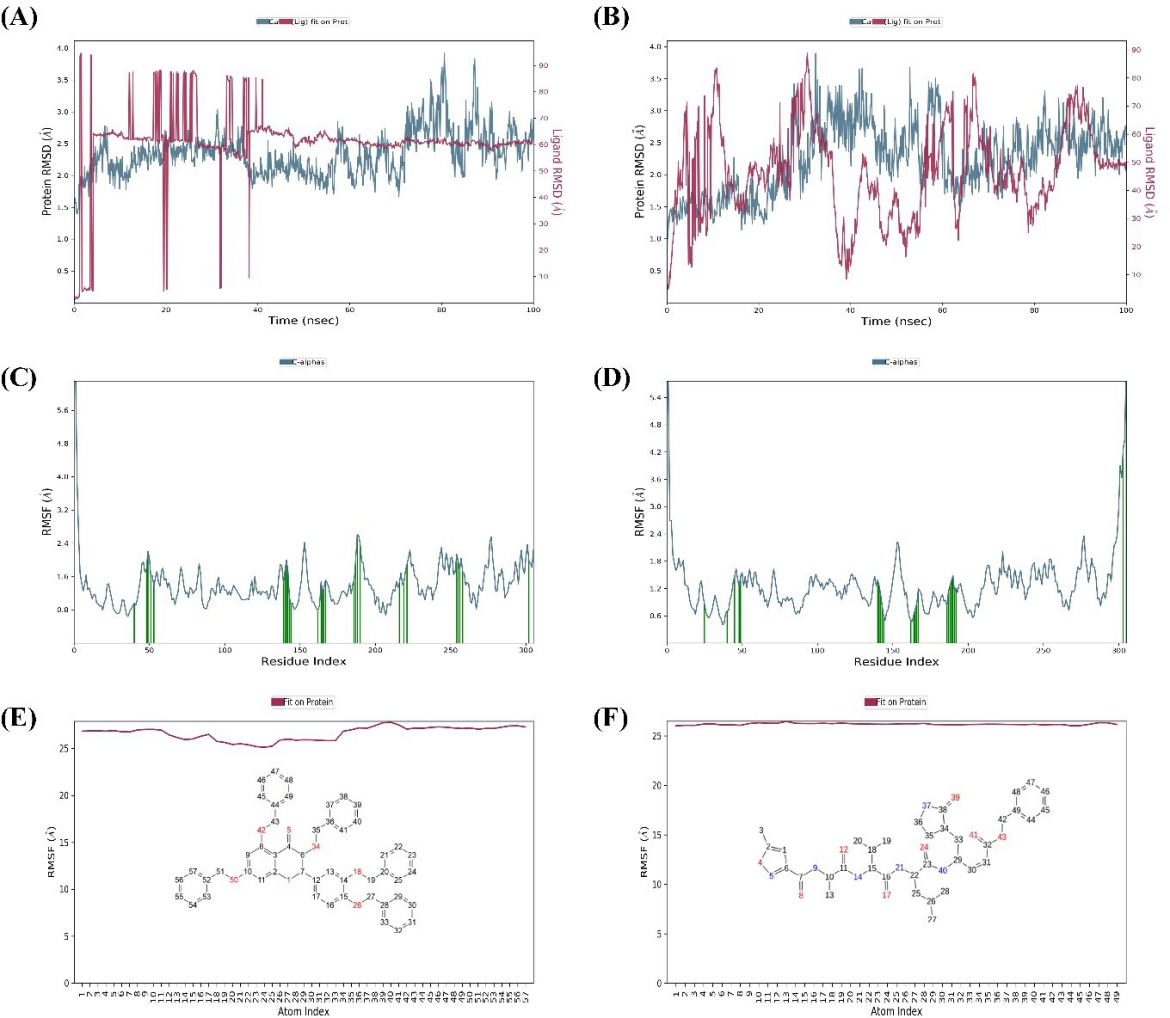
Protein-ligand RMSD trajectory, protein RMSF plot, ligand RMSF of SARS-CoV-2-382 complex (A, C, E) and SARS-CoV-2-Native ligand complex (B, D, F).

MD simulations were carried out for 100 ns to study the conformational stability of the ligand 382 at the active binding pocket of SARS-CoV-2 Mpro. In other words, the protein-ligand complex’s stability was studied through biophysical techniques such as MD simulation. The MD simulation studies for 382 were compared to the results of the native ligand of 6LU7. The RMSD trajectory of the protein-ligand complexes, the RMSF plot of the proteins, and the RMSF plot of the ligands for 382 and the native ligand are given in Fig. 4.

#### SARS-CoV-2 Mpro-382 complex

The RMSD trajectory of the SARS-CoV-2 Mpro-382 complex is given in Fig. 4A. The protein RMSD trajectory is represented in blue, and the RMSD values are shown on the left *Y*-axis in Å units. The ligand RMSD trajectory is represented in red color, and the RMSD values are given on the right *Y*-axis in Å units. From the original frame (*i.e.,* the original binding pose of the ligand obtained from docking studies) of the SARS-CoV-2 Mpro-382 complex, the protein RMSD started to fluctuate from around 1.5 Å at 0 ns. The protein RMSD trajectory gradually increases to and above 2.5 Å at around 10 ns. After this, the protein RMSD trajectory decreases to about 2.0 Å at around 15 ns. Then, between 15 ns and 40 ns, the protein RMSD trajectory fluctuates between 2.0 Å and 2.5 Å. After 40 ns until around 55 ns, the protein RMSD fluctuates at around 2.0 Å. After 55 ns to 70 ns, the protein RMSD fluctuates between 2.0 Å and 3.0 Å. From 70 ns to around 90 ns, the protein RMSD trajectory showed large fluctuations between 2.0 Å and 4.0 Å. From 90 ns onwards, the RMSD trajectory showed signs of decreasing fluctuations till the end of the simulation. Overall, majority of the protein RMSD fluctuates between 2.0 Å and 3.0 Å. The RMSD trajectory of the protein suggest the stability of the protein during the 100 ns MD simulation. The ligand RMSD trajectory showed large fluctuations between 10 Å and 90 Å at the beginning of the MD simulation. This pattern of large fluctuations for the ligand RMSD trajectory continued till 40 ns. However, from 40 ns onwards, the ligand RMSD trajectory stabilizes at around 60 Åtill the end of the simulation. The RMSD trajectory of the ligand suggests that from the original binding pose, the ligand might be trying to find a more suitable binding pose during the simulation and towards the end of the simulation, it has found a more suitable binding pose as the trajectory stabilizes at around 60 Å till 100 ns. The ligand RMSD is measured against the protein backbone which is taken as the reference.

**Table 1:** Binding energies of the best quercetin derivative, the standards, and the native ligand.

**Compound**	**Binding energy (kcal/mol)**
Ledipasvir	−10.59
Cobicistat	−9.46
Native	−8.743
382 (quercetin derivative)	−11.104

**Figure 5:**
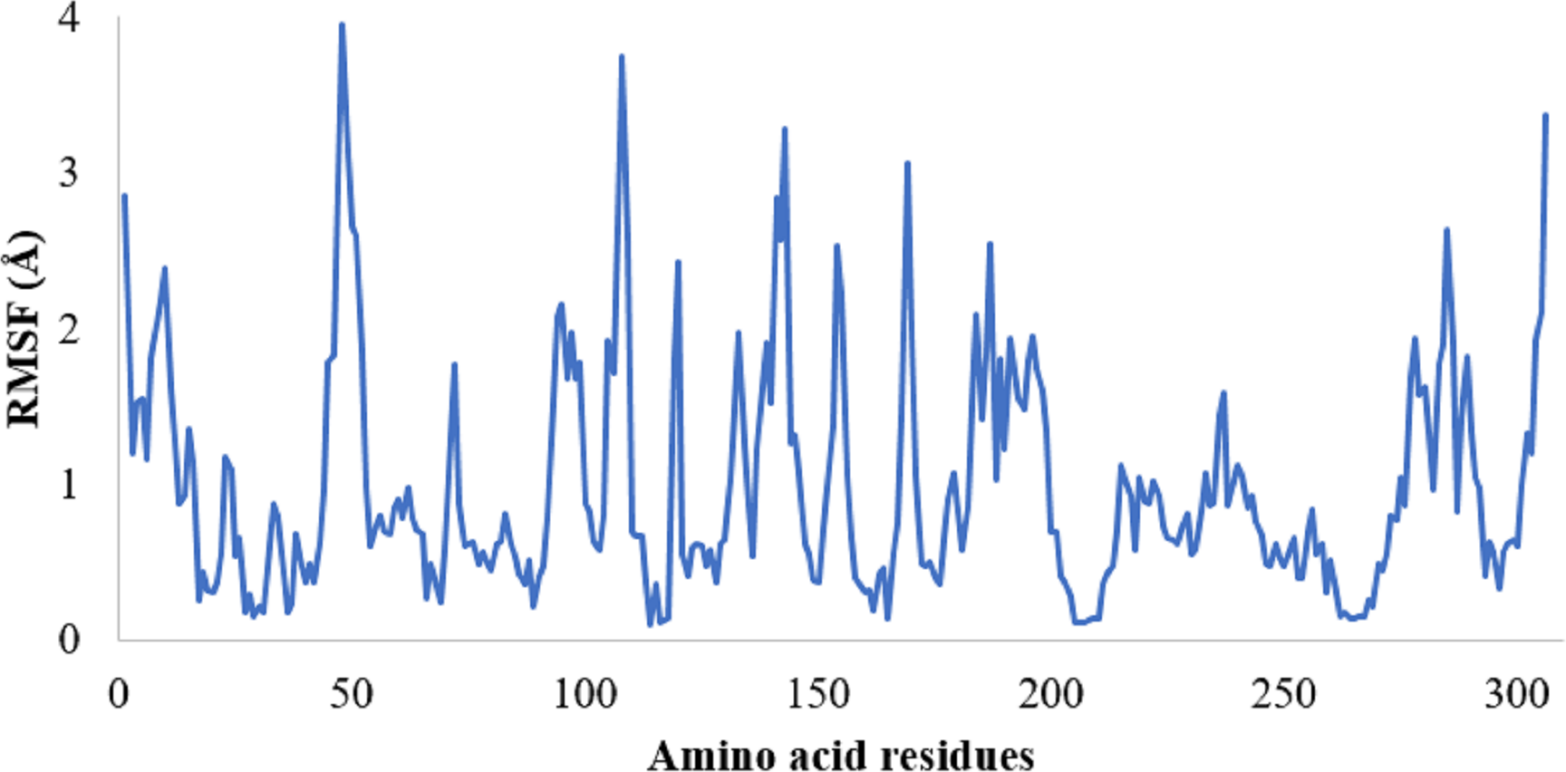
RMSF of the SARS-CoV-2 Mpro residues in its free state.

The protein RMSF plot of SARS-CoV-2 Mpro in the SARS-CoV-2 Mpro-382 complex is given in Fig. 4C. The green bars in the protein RMSF plot represent the amino acids interacting with the ligand. The terminal amino acid residues have a high RMSF value. On average, the protein RMSF value of the amino acid residues stayed below 2.0 Å. Most of the interacting amino acid residues also showed an RMSF value lower than 2.0 Å except for a few amino acids with residue numbers at around 180. The results of the RMSF of the protein free state that was studied with the CABS flex server 2.0 is given in Fig. 5. The terminal amino acid residues of the protein free state also showed a higher RMSF value in comparison to other residues. Also, a side by side comparative analysis of the RMSF plot between the SARS-CoV-2 Mpro-382 complex and the protein free state suggests a lower RMSF value associated with the SARS-CoV-2 Mpro-382 complex. The fluctuation in the protein RMSF seems to be caused by a loop region in the protein as the SARS-CoV-2 Mpro-382 complex tend to show a lower RMSF fluctuation than the protein free state. Based on this, the protein RMSF plot indicates the stability of each amino acid residues during the 100 ns MD simulation. The ligand RMSF plot of 382 in the SARS-CoV-2 Mpro-382 complex is given in Fig. 4E. On average, the ligand RMSF plot indicates that each atom of 382 has an RMSF value of above 25 Å. The ligand RMSF plot of 382 suggests that the ligand was constantly changing its binding pose in search of a more stable binding pose.

**Table 2:** Bond type and bond length of 382 in complex with 6LU7.

No.	Residue	Bond type	Bond length
1.	LYS5	Conventional hydrogen bond	2.22 Å
2.	LYS5	Conventional hydrogen bond	3.04 Å
3.	GLN	Carbon hydrogen bond	3.58 Å
4.	ASP289	Pi-Anion	3.34 Å
5.	LYS137	Pi-Sigma	3.60 Å
6.	LYS137	Pi-Alkyl	4.54 Å
7.	LYS5	Pi-Alkyl	5.34 Å
8.	PHE291	Pi-Pi Stacked	3.95 Å
9.	TYR126	Pi-Pi Stacked	4.04 Å

**Figure 6:**
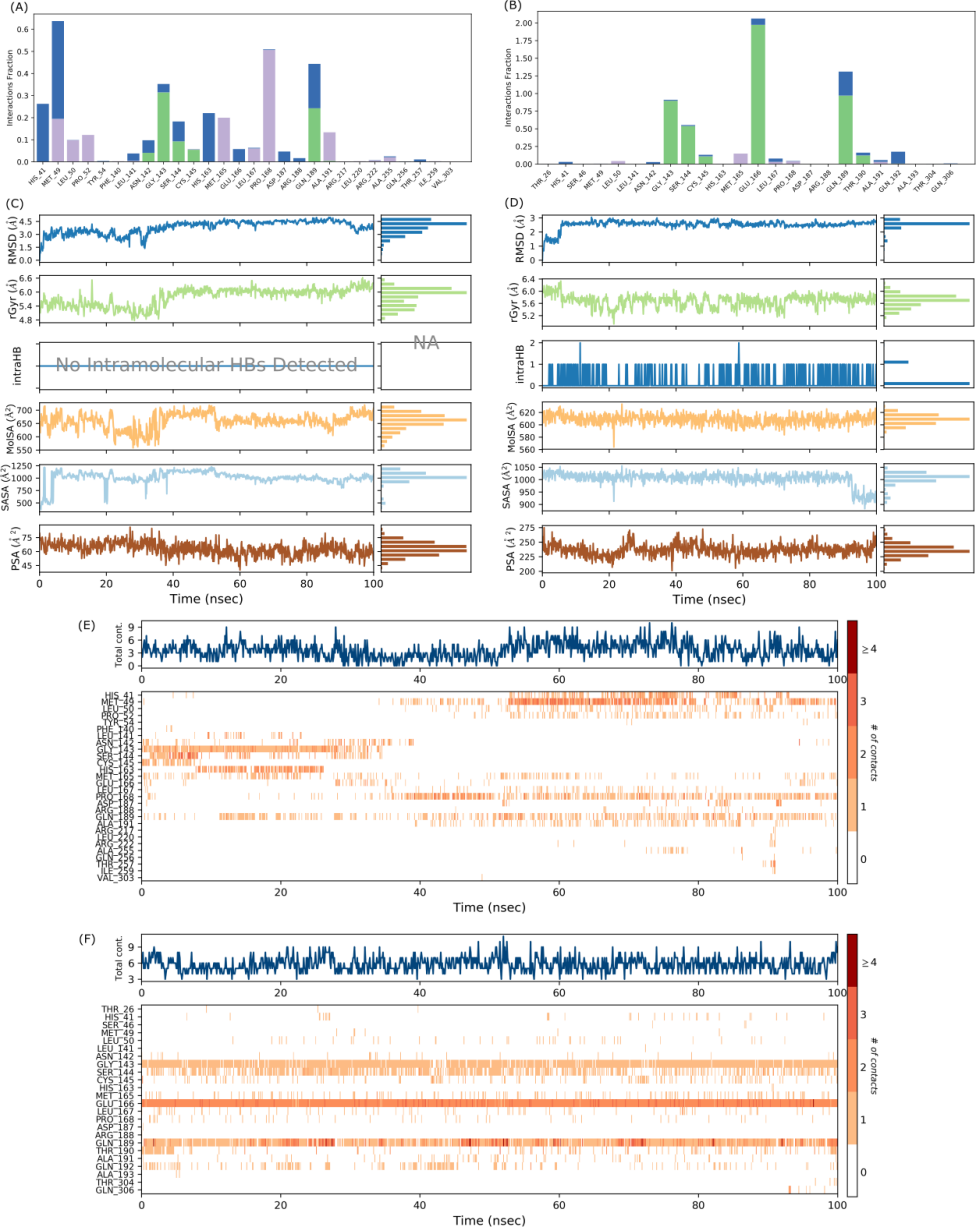
Protein-ligand interaction diagram, interaction timeline, ligand properties of SARS-CoV-2 Mpro-382 complex (A, C, E) and SARS-CoV-2 Mpro-native ligand complex (B, D, F).

The protein-ligand contact for the SARS-CoV-2 Mpro-382 complex during the 100 ns MD simulation is given in Fig. 6A. The interactions are further divided into four sub-types: hydrogen bonds, hydrophobic interactions, ionic interactions, and water bridges. The stacked bar charts are normalized throughout the trajectory; for example, a higher value of interaction fraction suggests that the specific interaction was adequately maintained during the 100 ns MD simulation. During the MD simulations, 382 formed conventional hydrogen bonds with ASN142, GLY143, SER144, CYS145, and GLN189. For the conventional hydrogen bonds, GLY143 showed the highest interaction fraction slightly higher than 0.3 followed by GLN189 (interaction fraction slightly below 0.3), SER144 (interaction fraction around 1), CYS145 (interaction fraction slightly higher than 0.05) and ASN142 (interaction fraction around 0.05). Also, 382 interacted with HIS41 through a water bridge (hydrogen-bonded protein-ligand interactions mediated by a water molecule). The timeline representation of the interactions and contacts (H-bonds, hydrophobic, ionic, and water bridges) is summarized in Fig. 6C. The top panel shows the total number of specific contacts the protein makes with the ligand throughout the trajectory. From the start of the simulation till around 30 ns, there were approximately five total contacts. Between 30 ns and 50 ns, the total contacts slightly decreased at around three. Between 50 ns and 80 ns, the total contacts were maintained at around six. After 80 ns till the end of the simulation, the total contacts fluctuate around three contacts. The higher number of total contacts was nine and the lowest total contact was approximately one. In each trajectory frame, the bottom panel displays which residues interact with the ligand. According to the scale on the right of the figure, certain residues make more than one particular contact with the ligand, which is indicated by a darker shade of orange (Velu et al., 2022). If we observe the intensity of the protein-ligand contacts in Fig. 6C for the first 40 ns of the MD simulation, the ligand does not seem to interact with half of the highlighted amino acids. After 40 ns, 382 showed a slight change in the pattern of ligand contacts. In Fig. 4A, we have already explained that the fluctuations of the ligand RMSD might indicate an unstable binding pose of the ligand. These two observations indicate that 382 was unstable at the active binding pocket for the first 40 ns of the MD simulation but remained stable thereafter.

**Figure 7:**
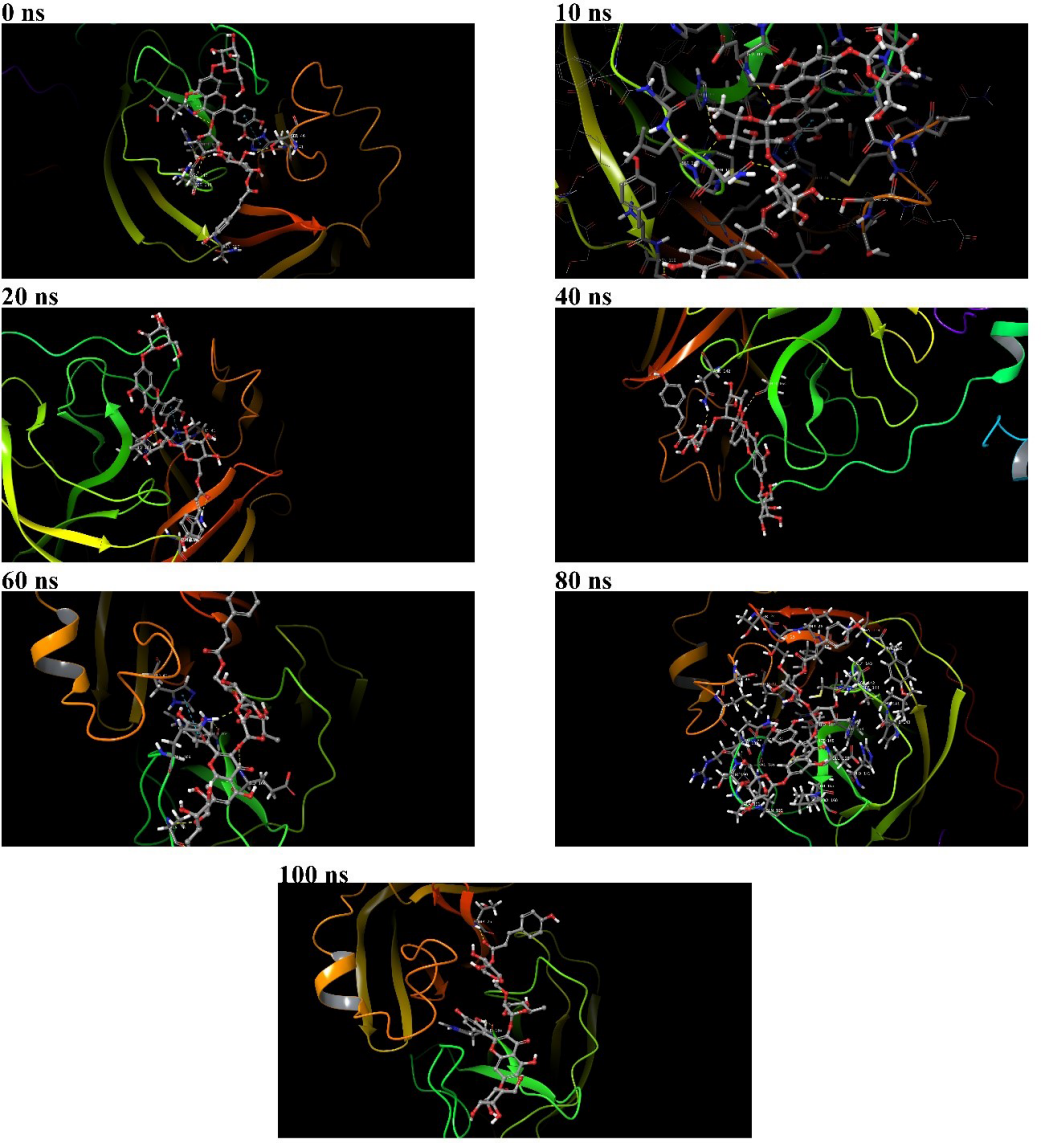
Binding poses of 382 at the active binding pocket of SARS-CoV-2 Mpro at different time points, namely 0 ns, 10 ns, 20 ns, 40 ns, 60 ns, 80 ns, and 100 ns.

Different ligand properties of 382 in the SARS-CoV-2 Mpro-382 complex, namely the RMSD, radius of gyration (rGyr), intramolecular hydrogen bond, molecular surface area (MolSA), solvent accessibility surface area (SASA), and polar surface area (PSA) were studied throughout the 100 ns MD simulations (Fig. 7E). The ligand RMSD fluctuated between 1.5 Å and 4.5 Å. Ultimately, the equilibrium was attained after 50 ns at around 4.0 Å. In this case, the ligand RMSD was measured with with respect to the ligand reference conformation (typically the first frame is used as the reference and it is regarded as time *t* = 0). The rGyr fluctuated between 4.8 Å and 6.6 Å, and the equilibrium was attained after 50 ns at around 6.0 Å. There was no intramolecular hydrogen bond within the 382 molecules. The MolSA fluctuated between 600 Å^2^ and 700 Å^2^, and the equilibrium was attained after 50 ns at around 650 Å^2^. A series of sharp fluctuations resulted in the SASA of 382 fluctuating between 500 Å^2^ and 1250 Å^2^, and the equilibrium was attained at around 1000 Å^2^ after 50 ns. The PSA of 382 fluctuates between 45 Å^2^ and 75 Å^2^, and the equilibrium was acquired at approximately 60 Å^2^.

During the 100 ns MD simulation, 382 tries to stabilize itself at the active binding pocket by finding the most suitable binding pose. Due to this, 382 tends to change its conformation at the active binding pocket, and this can be directly observed from the ligand RMSD of 382 in Fig. 4A. Also, it can be observed that as 382 tries to stabilize itself at the active binding pocket, it interacted with different amino acid residues in the process (Figs. 6A and 6B). The other binding poses of 382 at the active binding pocket of SARS-CoV-2 Mpro at different time points, namely 0 ns, 10 ns, 20 ns, 40 ns, 60 ns, 80 ns, and 100 ns, are given in Fig. 7.

#### SARS-CoV-2 Mpro-native ligand complex

The RMSD trajectory of the SARS-CoV-2 Mpro-native ligand complex is given in Fig. 4B. The protein RMSD is represented in blue with its RMSD value given in Å units on the left *Y*-axis while the ligand RMSD is represented in red with its RMSD value given in Å units on the right *Y*-axis. The RMSD trajectory of the protein originated from 1.0 Å at 0 ns and gradually increased towards 3.0 Å at around 30 ns. From 30 ns to approximately 60 ns, the protein RMSD trajectory decreases between 2.0 Å and 2.5 Å. From then on, the protein RMSD trajectory stabilizes around 2.5 Å till the end of the simulation. The RMSD trajectory of the protein suggest the stability of the protein during the 100 ns MD simulation. The ligand RMSD trajectory originates around 10 Å and then shows a sharp rise in RMSD value to about 80 Å. This fluctuation pattern was observed till 90 ns of the simulation, wherein the ligand RMSD showed a sharp rise and fall. From 90 ns till the end of the simulation, the ligand RMSD trajectory fluctuates around 50 Å. The RMSD trajectory of the ligand suggests that from the original binding pose, the native ligand might be trying to find a more suitable binding pose during the simulation and towards the end of the simulation, it has found a slightly better binding pose as the trajectory fluctuates at around 50 Å from 90 ns until 100 ns. Overall, the ligand RMSD of 382 (Fig. 4A) suggests a greater stability at the active binding pocket when compared to the RMSD of the native ligand (Fig. 4B).

The protein RMSF plot of SARS-CoV-2 Mpro in the protein-ligand complex is given in Fig. 4D. The terminal amino acid residues showed a high RMSF value greater than 2.0 Å. The N-terminal of the protein has a loop conformation and hence showed high RMSF fluctuations (Arya et al., 2021). However, all the other amino acid residues showed a lower RMSF value than 2.0 Å. The vertical green bars representing the protein’s interacting amino acid residues all showed an RMSF value lower than 2.0 Å. The protein RMSF plot indicates the stability of each amino acid residues during the 100 ns MD simulation. The ligand RMSF of the native ligand in the protein-ligand complex is given in Fig. 4F. All the atoms in the native ligand can be correlated with the atom index provided on the lower *x*-axis, and their corresponding RMSF value can be observed on the left *Y*-axis. The atoms of all the native ligands showed an RMSF value higher than 25 Å. The ligand RMSF plot of the native ligand suggests that the ligand was constantly changing its binding pose in search of a more stable binding pose. This indicates that the native ligand was not stable at the active binding pocket of the protein.

The interaction chart between SARS-CoV-2 Mpro and the native ligand is given in Fig. 6B. The interaction fraction is given on the left *Y*-axis, while the interacting amino acid residues are given on the lower *X*-axis. During the 100 ns MD simulations, GLY143, SER144, CYS145, GLU166, GLN189, and THR190 formed hydrogen bonds with the native ligand. HIS41 of the catalytic dyad also interacted with the native ligand through a water bridge. The stacked bar charts are normalized throughout the trajectory; for example, a higher value of interaction fraction suggests that the specific interaction was adequately maintained during the 100 ns MD simulation. The timeline representation of the interactions and contacts is summarized in Fig. 6D. The top panel shows the total number of specific contacts the protein makes with the ligand. The bottom panel shows the interacting residues in each trajectory frame. A darker shade of orange represents residues that make more contact.

Different properties of the native ligand that were studied throughout the 100 ns MD simulations are given in Fig. 6F. The ligand RMSD fluctuated between 1.0 Å and 3.0 Å, and the equilibrium was attained after 5 ns at around 3.0 Å. In this case, the ligand RMSD is measured with reference to its original frame. This explains the lower RMSD value in comparison to the high RMSD value in Fig. 4B where the ligand RMSD value was measured with the protein backbone taken as the reference. In general, the rGyr fluctuated between 5.2 Å and 6.4 Å, and the equilibrium was achieved after 70 ns at around 5.6 Å. One intramolecular hydrogen bond was observed for the native ligand. The MolSA fluctuated between 580 Å^2^ and 620 Å^2^, and the equilibrium was observed at around 610 Å^2^. The SASA of the native ligand fluctuates between 900 Å^2^ and 1,050 Å^2^, a decrease from 1,000 Å^2^ to 900 Å^2^ was observed at around 90 ns, and the equilibrium was attained at around 1,000 Å^2^. The PSA of the native ligand fluctuates between 200 Å^2^ and 275 Å^2^ throughout the simulation and the equilibrium was reached at about 230 Å^2^. Considering the frequency and degree of trajectory fluctuations, and the values given on the right *Y*-axis of each parameter (see Figs. 6E and 6F), a preliminary comparative analysis suggest that 382 showed higher signs of stability than the native ligand.

### MM-GBSA BFE calculations

**Figure 8:**
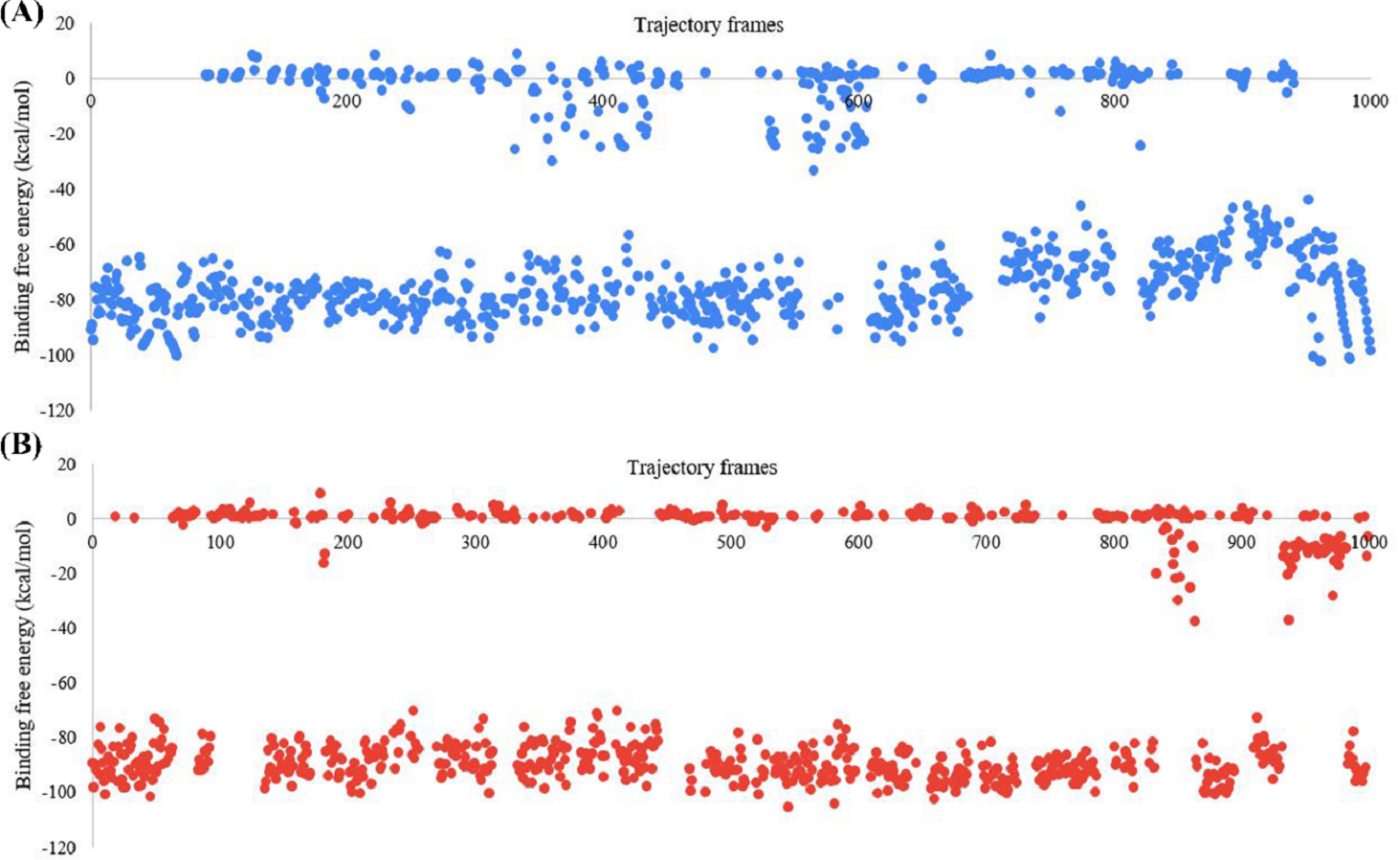
Graph for the estimated MM-GBSA values (left *Y*-axis) *vs* time (ns) (top *X*-axis) for (A) 382 and (B) the native ligand. The trajectory frames are a representative of the time in the MD simulation. For example, 1st trajectory frame = 0.1 ns, 2nd trajectory f.

The BFE of the SARS-CoV-2 Mpro-382 complex and the SARS-CoV-2 Mpro-native ligand complex was studied with the MM-GBSA approach. The BFE of the SARS-CoV-2 Mpro-382 complex and SARS-CoV-2 Mpro-native ligand complex in each trajectory frame is given in Fig. 8. The BFE value is provided on the left *Y*-axis, and the individual trajectory frames are given on the *X*-axis. The trajectory frames (*n* = 1, 000) correspond to the time of the MD simulation. For instance, the BFE in the first frame indicates the BFE at 0.1 ns of the 100 ns MD simulation. The average BFE of both protein-ligand complexes is shown in Table 3. The average BFE of the SARS-CoV-2 Mpro-382 complex during the 100 ns MD simulation was calculated to be −54.0 kcal/mol. The average BFE of the SARS-CoV-2 Mpro-native ligand complex during the 100 ns MD simulation was estimated to be −52.8 kcal/mol. The SARS-CoV-2 Mpro-382 complex has a slightly lower BFE than the SARS-CoV-2 Mpro-native ligand complex. On average, the protein-382 complex has a lower BFE than the protein-native ligand complex by a margin of 1.6 kcal/mol. This slight difference in the average BFE is enough to justify that the protein-382 complex is slightly more stable than the protein-native ligand complex.

**Table 3:** Average MM-GBSA BFE of protein-ligand complexes.

**Protein-ligand complex**	**ΔG (kcal/mol)**
SARS-CoV-2 Mpro-382 complex	−54.02045
SARS-CoV-2 Mpro-native ligand complex	−52.7576

### *In-silico* ADMET studies

The ADMET properties of 382 and the native ligand is given in Table 4. For both 382 and the native ligand, the formula, molecular weight, number of heavy atoms, number of aromatic heavy atoms, fraction Csp3, number of rotatable bonds, number of H-bond acceptors/donors, molar refractivity, and topological polar surface area is given in Table 3. The consensus log P of 382 and the native ligand are 8.29 and 2.69, respectively. The native ligand and 382 will have low gastrointestinal absorption and they will not permeate the blood brain barrier. The action of p-glycoproteins might remove the native ligand from the cell while it will not impact 382. Out of the studied hepatic enzymes, the native ligand might interact with CYP3A4 inhibitor while 382 was predicted not to interact with any hepatic enzymes. The skin permeability rate of 382 and the native ligand are −3.63 cm/s and −8.07 cm/s, respectively. Against the studied bioavailability parameters, 382 showed a total of 12 violations (two against the Lipinski rule of five; four against the Ghose filer and Muegge filter, one against the Veber filter and Egan filter) while the native ligand showed a total of 11 violations (two against the Lipinski rule of five and Veber filter, three against the Ghose filter and Muegge filter, one against the Egan filter). However, both 382 and the native ligand has a bioavailability score of 0.17. In leadlikeness category, 382 showed three violations while the native ligand showed only two violations. However, 382 will be synthesized more easily with a slightly lower synthetic accessibility score of 6.24 as compared to 6.43 for the native ligand. Both 382 and the native ligand showed no signs of toxicity against the studied parameters.

**Table 4:** ADMET properties of 382 in comparison to the native ligand.

**Properties**	382	**Native ligand**
Formula	C_50_H_42_O_7_	C_35_H_48_N_6_O_8_
Molecular weight	754.86	680.79
Number of heavy atoms	57	49
Number of aromatic heavy atoms	42	11
Fraction Csp3	0.14	0.51
Number of rotatable bonds	16	22
Number of H-bond acceptors	7	9
Number of H-bond donors	0	5
Molar refractivity	219.8	184.13
Topological polar surface area	72.45	197.83
Consensus Log P	8.29	2.69
Water solubility	Insoluble	Poorly soluble
Gastrointestinal absorption	Low	Low
Blood brain barrier permeant	No	No
P-glycoprotein substrate	No	Yes
CYP1A2 inhibitor	No	No
CYP2C19 inhibitor	No	No
CYP2C9 inhibitor	No	No
CYP2D6 inhibitor	No	No
CYP3A4 inhibitor	No	Yes
Skin permeability log Kp (cm/s)	−3.63	−8.07
Lipinski violations	2	2
Ghose violations	4	3
Veber violations	1	2
Egan violations	1	1
Muegge violations	4	3
Bioavailability Score	0.17	0.17
Leadlikeness violations	3	2
Synthetic Accessibility	6.24	6.43
Predicted LD_50_	2000 mg/kg bodyweight	4000 mg/kg bodyweight
Toxicity class	4	5
Hepatotoxicity	Inactive	Inactive
Carcinogenicity	Inactive	Inactive
Immunogenicity	Inactive	Inactive
Mutagenicity	Inactive	Inactive
Cytotoxicity	Inactive	Inactive

## Discussion

MDSS is one of the most affordable and commonly used *in-silico* techniques. MDSS uses a scoring function to rank a compound’s binding pose. Among different binding poses generated from MDSS, the first pose is consistently ranked as the best for the ligand against a particular protein. As such, MDSS can be used to study the binding affinity, binding pose, and molecular interactions of a ligand with a protein. In the drug discovery process, MDSS is used to screen a compound library of many molecules to identify promising inhibitors/stimulators against a target protein (Zothantluanga, 2021). Among hundreds of quercetin derivatives that were virtually screened, compound 382 was observed as the most promising molecule to inhibit the Mpro of SARS-CoV-2. This observation was made based on the binding affinity of compound 382 (−11.1 kcal/mol) towards the active binding pocket of SARS-CoV-2 Mpro. In addition to having low docking score, compound 382 interacted with LYS5, TYR126, GLN127, LYS137, ASP289, PHE291, ARG131, SER139, GLU288, and GLU290 through conventional hydrogen bonds, hydrophobic interactions, electrostatic interactions, and van der Waals interactions. Compound 382 needs to form conventional hydrogen bonds because hydrogen bonds are directly associated with protein catalysis, molecular recognition, regulation of protein structure, and drug resistance. If there is any deletion, addition, or substitution of any interacting amino acid of a protein that takes part in the formation of a hydrogen bond, it will alter the outcome of the protein-ligand interaction. So, a higher number of conventional hydrogen bond formations between a protein and ligand is always preferable to a smaller number (Pasala et al., 2022a).

MDSS is often combined with *in-vitro* and *in-vivo* studies. Even though MDSS is a fast and reliable *in-silico* technique, the results obtained from MDSS often need validation. The docking protocol used for MDSS can be validated through the re-docking of the native ligand followed by calculating the RMSD value between the superimposed structure of the re-docked native ligand and the original co-crystallized complex (Zothantluanga et al., 2021a). On the other hand, the results of MDSS can also be validated with *in-vitro* studies. Hundreds of semi-synthetic derivatives and synthetic compounds are virtually screened with MDSS, after which the synthesized compounds’ potency is evaluated *in-vitro* (Rath et al., 2021). Sometimes, MDSS is combined with *in-vivo* studies to study the molecular interactions between compounds and biomarkers such as interleukins or tumor necrosis factors  (Pasala et al., 2022b; Pasala et al., 2022a). However, if a research work comprises only *in-silico*, MDSS studies must be validated with MD simulations for at least 100 ns. Several researchers have validated the results of MDSS with MD simulations wherein the stability of a protein-ligand complex is observed (Prajapati et al., 2021; Rudrapal et al., 2021). As the present work on quercetin derivatives is based on *in-silico*, we have decided to validate the MDSS studies with MD simulations to check the conformational stability of a ligand at the active binding site of the protein.

In the present study, MD simulation was carried out for the SARS-CoV-2 Mpro-382 complex and the SARS-CoV-2 Mpro-native ligand complex. In both protein-ligand complexes, the stability was studied using parameters such as RMSD, RMSF, protein-ligand contacts, and ligand properties. For small globular proteins, a fluctuation in the RMSD not more than 2 Å is acceptable to confer stability status for a protein (Rudrapal et al., 2022b). On the other hand, a ligand RMSD value slightly higher than the observed protein RMSD value is acceptable. However, if the ligand RMSD value’s fluctuation is too high compared to the protein RMSD value, the ligand is trying to find another binding pose with higher stability (Umar et al., 2022; Zothantluanga et al., 2022). In the present study, the proteins in the SARS-CoV-2 Mpro-382 complex and the SARS-CoV-2 Mpro-native ligand complex showed stable conformation. However, both ligands in both protein-ligand complexes are trying to find a different pose with higher stability than the initial pose. A similar result in which a ligand attempted to find a more stable pose was also reported (Zothantluanga et al., 2022). The overall RMSD analysis of the protein-ligand complex indicates that the stability of the protein in the SARS-CoV-2 Mpro-382 complex and the SARS-CoV-2 Mpro-native ligand complex. However, the ligand RMSD indicates that 382 was more stable at the active binding pocket of SARS-CoV-2 Mpro than the native ligand.

The RMSF of the protein and the ligand were studied through an all-atom MD simulation. The terminal amino acids generally show a higher RMSF value when compared to other amino acid residues. In an ideal case, most amino acids should show an RMSF value lower than 2 Å. A protein’s interacting amino acid residues must show an RMSF value lower than 2 Å. In both protein-ligand complexes of 382 and the native ligand, the amino acids of both complexes showed RMSF value lower than 2 Å. These RMSF results are indicative of a stabilized protein. However, the ligand RMSF value showed a different picture. For the native ligand and 382, each compound atom has an RMSF value as high as 25 Å. Just as the protein RMSF result corroborated the protein RMSD, the ligand RMSF supported the ligand RMSD result. From the protein-ligand contacts, it can be observed that 382 and the native ligand interacted with many amino acids that were not seen in the results of MDSS. This could only happen if the ligand changes its position within the active binding site. By changing its pose at the active binding site, the ligand will form interactions with other amino acids. The native ligand and 382 maintained significant interactions with the protein throughout the 100 ns MD simulation. It can be deciphered that the ligand RMSD, ligand RMSF, and protein-ligand contacts are well correlated with each other. Different ligand properties such as RMSD, rGyr, MolSA, SASA, and PSA were also studied. The rGyr determines the compactness of a protein (Rudrapal et al., 2022a).

Once the stability of a protein-ligand complex is ascertained through MD simulation studies, it is essential to validate MDSS with MM-GBSA BFE calculations further. The MM-GBSA BFE approach calculates the average amount of energy released during the 100 ns MD simulations. Our previous discussion has elaborated on the formation of different interactions between SARS-CoV-2 Mpro, the native ligand, and 382. The occurrence of protein-ligand contacts resulted in the release of energies. This is measured with the MM-GBSA BFE approach  (Khater et al., 2021). One ns is made up of a ten smaller units, for example, 0.1, 0.2, 0.3, 0.4, and so on. Each nanosecond of the 100 ns was split into 1000 frames by a thermal script of Schrodinger. The BFE of the protein-ligand complexes in each frame was calculated with the MM-GBSA approach. In the present study, we found that the SARS-CoV-2 Mpro-382 complex has a slightly lower BFE than the SARS-CoV-2 Mpro-native ligand complex. In MDSS, 382 showed a better docking score than the native ligand. The MM-GBSA approach validates the results of MDSS as the protein-382 complex has a better BFE than the protein-native ligand complex. The results of the MD simulations (such as the RMSD of the protein and the ligand) also suggested that 382 showed higher signs of stability with the protein than the native ligand. Based on the MDSS, MD simulations, and MM-GBSA BFE calculations, it can be stated that 382 has a higher potential to inhibit the Mpro of SARS-CoV-2 than the native ligand.

From the *in-silico* ADMET studies, both compound 382 and the native ligand were observed to be associated with bioavailability issues. It was predicted that both 382 and the native ligand will not be absorbed from the gastrointestinal tract nor it would permeate the blood brain barrier. However, the consensus Log *P*_o/w_ value of 382 was found to be a higher than the native ligand. The higher the log *P* value of a compound, the higher is the hydrophobicity of a compound (Umar et al., 2022). Based on its log *P* value alone, compound 382 might be able to permeate the blood brain barrier and other physiological membranes better than the native ligand (Banks, 2009). However, from the chemical structure and the molecular weight, we can observe that both compound 382 and the native ligand are very bulky chemical molecule. The size of compound 382 and the native ligand might be responsible for the predicted low bioavailability. Moreover, bioavailability filters such as Lipinski’s rule of five, Ghose filter, Veber filter, Muegge filter, and Egan filter also reported some 12 and 11 violations compound 382 and the native ligand, respectively  (Lipinski, 2004; Ghose, Viswanadhan & Wendoloski, 1999; Veber et al., 2002; Muegge, Heald & Brittelli, 2001; Egan, Merz & Baldwin, 2000). On the bright side, both compound 382 and the native ligand passed all toxicity test. For a compound to be considered as potential candidate for further studies, it should not show signs of toxicity (Zothantluanga et al., 2021a). Regarding the bioavailability issues for compound 382, we suggest the use of novel drug delivery systems for natural compounds such as phytosome and herbosome (Pasala et al., 2022b; Rahman et al., 2020). Moreover, compound 382 can also be encapsulated with polymers to enhance its bioavailability (Pachuau et al., 2021; Sriwidodo et al., 2020; Sriwidodo et al., 2022; Umar, 2022; Umar et al., 2021; Umar et al., 2020; Fadhila, Sriwidodo & Chaerunisa, 2022). It is worthy to note that the native ligand is a peptide. Therefore, it will be associated with more issues in the manufacturing step as compared to conventional drugs. Moreover, peptide has a low *in vivo* stability due to enzymatic or endocytosis receptor mediated degradation. Considering these factors, 382 is favoured over the native ligand for further studies and processing.

Natural products have always been a reservoir of bioactive molecules for treating various diseases. Many natural products have shown potent activity against SARS-CoV-2. An extract of *Vitis vinifera* containing many flavonoids was reported to exhibit potent activity against SARS-CoV-2 *in-vitro* (Zannella et al., 2021). In another study, a terpenoid known as oridonin was reported to inhibit the *in-vitro* replication of SARS-CoV-2. The study also reported that oridonin binds and inhibits the non-structural protein-9 of SARS-CoV-2 (Littler et al., 2021). Recently, a traditional herbal formulation of Africa consisting of four medicinal plants (*Clerodendrum glabrum E. Mey.* Lamiaceae, *Gladiolus dalenii* van Geel, *Rotheca myricoides* (Hochst.) Steane & Mabb, and *Senna occidentalis* (L.) Link) was tested for its potency against SARS-CoV-2. The *in-vitro* study found that 0.005 mg/ml of the herbal formulation prevented >90% of SARS-CoV-2 infection and showed an IC_50_ value of about 0.01 mg/ml. MDSS studies also revealed that the compounds present in the herbal formulation showed high binding affinity towards the spike protein of SARS-CoV-2 (Matsabisa et al., 2022). Many other studies have also found that natural compounds are potential inhibitors of SARS-CoV-2 (Muegge, Heald & Brittelli, 2001; Egan, Merz & Baldwin, 2000; Rahman et al., 2020; Pachuau et al., 2021; Sriwidodo et al., 2020). A study carried out by Ibrahim, Abdelrahman & Hegazy (2020) used a combination of molecular docking, MD simulations and MM-GBSA calculations to identify cobicistat as a potential inhibitor of SARS-CoV-2 Mpro (Sriwidodo et al., 2022). In the present study, we have used a similar approach to generate enough *in-silico* evidence to consider the quercetin derivative 382 as a promising inhibitor of SARS-CoV-2 Mpro.

## Conclusion

Among the 470 quercetin derivatives, we found compound 382 as the most promising molecule to inhibit the replication of SARS-CoV-2 by binding and inhibiting the Mpro. In MDSS, compound 382 showed a better docking score (higher binding affinity) than the native ligand. From the MDSS, we observe that compound 382 showed a good protein-ligand interactions. As MDSS revealed that 382 has a better docking score than the native ligand, we carried out MD simulations for 100 ns to check the stability of the ligands at the active binding pocket. In MD simulations, the ligand RMSD trajectories suggested that 382 will be more stable at the active binding pocket than the native ligand. The interaction fraction, total contacts, and ligand properties of 382 are equivalent to that of the native ligand. In addition, the MM-GBSA BFE value of 382 was slight lower than the native ligand. Based on this, we conclude that compound 382 has a better potential to inhibit SARS-CoV-2 Mpro than the native ligand. However, further studies such as *in-vitro* assays are recommended to confirm its *in-silico* potency.

##  Supplemental Information

10.7717/peerj.14915/supp-1 Supplemental Information 1 Docking files

10.7717/peerj.14915/supp-2 Supplemental Information 2 Protein Dataset

10.7717/peerj.14915/supp-3 Supplemental Information 3 Ligands Dataset

## Additional Information and Declarations

### Data Availability

The following information was supplied regarding data availability: The protein data is available at RSCB Protein Database: 6LU7. https://www.rcsb.org/structure/6LU7. The other data is available in the Supplemental Files.
